# Sleep and the Speed of Progression from Mild Cognitive Impairment to Dementia

**DOI:** 10.1093/geroni/igaf122.1841

**Published:** 2025-12-31

**Authors:** Ning Zhang, Lichuan Ye, Jack Johnson

**Affiliations:** Northeastern University, Boston, Massachusetts, United States; Northeastern University, Boston, Massachusetts, United States; Northeastern University, Boston, Massachusetts, United States

## Abstract

**Background:**

The factors contributing to the progression from mild cognitive impairment (MCI) to Alzheimer’s Disease Related Dementia (ADRD) remain unclear. Increasing evidence suggests sleep disturbances are a significant risk factor for the rate of developing ADRD. This study aims to explore how sleep issues affect the speed of cognitive decline from MCI to ADRD.

**Methods:**

This secondary analysis used data from the Health Retirement Survey (HRS, 2006-2020), a biennial survey of Americans aged 50 or older. Sleep disturbances included self-reported sleep complaints and short sleep duration (< 6 hours). The speed of cognitive decline was measured by the number of years an individual with MCI remained in the MCI stage before transitioning to ADRD, with assessments conducted every two years. MCI was evaluated using standardized cognitive test scores. Joint regression analyses, including logistic and survival analyses, were used to determine the association between sleep disturbances and cognitive progression from MCI to ADRD.

**Results:**

Among 5,228 MCI participants, 45% reported sleep disturbances. Those with sleep complaints (OR = 1.14) or short sleep duration (OR = 1.37) were more likely to develop ADRD. Among those who developed ADRD, sleep disturbances predicted a faster transition from MCI to dementia—2 years for sleep complaints and 4 years for short sleep duration.

**Conclusions:**

Sleep disturbances in the early stages of Alzheimer’s may indicate a faster cognitive decline. Addressing these disturbances through targeted interventions could help delay or prevent cognitive impairment in older adults.

